# The Restructuring of Wage-Setting Fields between Transnational Competition and Coordination

**DOI:** 10.1007/s11614-018-0298-6

**Published:** 2018-05-16

**Authors:** Susanne Pernicka, Vera Glassner, Nele Dittmar

**Affiliations:** Institut für Soziologie, Abteilung Wirtschafts- und Organisationssoziologie, Johannes Kepler Universität Linz, Altenberger Straße 69, 4040 Linz, Austria

**Keywords:** Field Theory, Pierre Bourdieu, Power Relations, Social Services, Sociological Neo-Institutionalism, Wage-Setting Institutions, Feldtheorie, Pierre Bourdieu, Machtrelationen, Soziale Dienste, Soziologischer Neo-Institutionalismus, Institutionen der Lohnfindung

## Abstract

This article addresses the restructuring of wage-setting institutions in social services in Austria and Germany and seeks to better understand the forces shaping their changes and continuities. Social services include a wide range of services such as labour market policies or elderly care provided by state, private for- and non-profit organisations. Despite similar pressures resulting from European and national politics of economic liberalisation and austerity and the emergence of transnational corporations and labour migration wage-setting in social services turned out to follow different institutional paths. This article expands conventional theorising by using a social field perspective to uncover the role of (institutionalised) power relations and conflict dynamics in shaping the wage-setting institutions. While existing institutions and particular power relations between regional and national actors helped create a common wage-setting field in Austrian social services, the interactions between national and regional, and, to a lesser extent, transnational field participants have led to fragmented and still contested institutions of wage setting in German social services.

## Zusammenfassung

Dieser Aufsatz behandelt die Restrukturierung von Institutionen der Lohnfindung in den Sozialen Diensten in Österreich und Deutschland und zielt auf ein besseres Verständnis der treibenden Kräfte von deren Wandel und Kontinuität. Soziale Dienste umfassen ein breites Spektrum von Dienstleistungen, wie arbeitsmarktpolitische Maßnahmen oder Altenpflege, und werden durch staatliche, privat-gemeinnützige oder profitorientierte Organisationen bereitgestellt. Trotz ähnlicher Herausforderungen durch europäische und nationale Liberalisierungs- und Sparpolitiken, transnationale Unternehmen und Arbeitsmigration haben sich die Lohnfindungsinstitutionen in den sozialen Diensten unterschiedlich entwickelt. Dieser Aufsatz schlägt eine, über konventionelle Theorien hinausgehende, soziale Feldperspektive vor, die (institutionalisierte) Machtrelationen und Konfliktdynamiken in der Beeinflussung von Lohnfindungsinstitutionen betont. Während existierende Institutionen und besondere Machtrelationen zwischen regionalen und nationalen Akteuren die Schaffung eines österreichischen Feldes der Lohnverhandlungen in den sozialen Diensten unterstützt haben, trugen regionale und nationale Akteure, und in einem geringeren Ausmaß auch transnationale Akteure zu fragmentierten und weiterhin umkämpften Institutionen der Lohnfindung in den deutschen sozialen Diensten bei.

## Introduction

Political programs of economic liberalisation and public austerity have sought to restructure the provision of social services and to open the various supplier and labour markets within the European Union (EU). The European Commission Directorate-General responsible for the internal market particularly wants to increase the number of commercial actors in the field of social services rather than shelter these services from internal market rules (Baeten 2007). Hence, (quasi) market competition, the emergence of large transnational corporations in certain subsectors, such as elderly care, and labour migration in mobile and domestic care are widely seen as having increased pressures on wages and working conditions in social services. Social services include a wide variety of services such as health and social care including labour market policies, youth work and child and elderly care provided by state, private for- and non-profit organisations. Since social services are personal services, their provision is largely tied to a specific location. While this makes transnational relocation of social services unlikely, market structures and employment relations in the social services sector have nevertheless been reconfigured over the last ten to fifteen years in Europe (Kroos and Gottschall 2012; Dahme and Wohlfahrt 2015). Countries like Austria and Germany that strongly rely on non-public actors to provide social services have particularly experienced profound changes in their employment relations and wage-setting institutions. However, their transformation has not been unidirectional in the sense of becoming more market oriented. Austria and Germany used to be compared as most similar cases in terms of industrial relations (Pernicka and Aust 2007); however, their very heterogeneous wage-setting institutions and employment relations in social services range from recently introduced forms of autonomous collective bargaining between employers’ associations and trade unions in Austria to highly fragmented and contested employment relations and wage-setting institutions in Germany.

This article addresses the structuring and restructuring of employment relations and wage-setting institutions in German and Austrian social services, excluding health services^1^, and seeks to better understand the forces shaping their changes and continuities. This analysis will particularly explore the evolution, maintenance or disruption of wage-setting institutions^2^ in varying arenas of collective bargaining. Our focus will be on these arenas, and not on the various subsectors of social services, because wage setting institutions are tied to different types of carrier organisations (i. e. public, private for-profit and private non-profit), who might be involved in various types of services (see below and Evans et al. 2012). This article considers historically created social structures, power relations and shared meanings (i. e. rules, norms and shared understandings) that shape employer-employee relations in different societies, as well as actors who have contributed to the (re)production or change of wage-setting institutions.

In theoretical terms, this article refers to different strands of literature, including historical institutionalism, sociological neo-institutionalism and Bourdieusian approaches that have contributed to the vibrant debate on the trajectories of institutional change and continuity. In recent years, two key conceptual innovations have evolved and spread across these scholarly analyses. The first development explains institutional change by emphasising the endogenous forces, such as institutional entrepreneurship, instead of the exogenous shocks or environmental shifts (Streeck and Thelen 2005; Zietsma and Lawrence 2010). The second innovation calls for a more conflict-oriented and power-sensitive analysis of institutional change and continuities (Mahoney and Thelen 2010; Kluttz and Fligstein 2016). These theoretical innovations challenge traditional, cross-country comparisons employed by many researchers in industrial relations, welfare state research and political economy. With notable exceptions (Anner et al. 2006; Marginson 2016), these scholars have tended to remain within conventional spatial “containers” like the nation state or have explored the economic and political effects of globalisation and Europeanisation on specific national capitalisms. For example, since the 1980s, institutional approaches in industrial relations and political economic research have focused on whether or not contemporary market pressures drive a convergence of national capitalisms towards a liberal model (Marginson and Sisson 2006; Thelen 2012). Scholars of comparative institutionalism generally assume that market internationalisation or European liberalisation politics have not completely levelled down national varieties of capitalism. Instead, different institutional trajectories and, thus, path dependencies play a role in shaping the responses of national actors, such as trade unions, employers and public policy actors, towards these challenges (Traxler et al. 2001; Gumbrell-McCormick and Hyman 2013). The primary focus on comparisons of national systems can be justified by the fact that nation states still largely shape employment relations, labour market and welfare state regulations; struggles over continuity and change mainly take place within national arenas. However, this article seeks to demonstrate that even social fields (e. g. collective wage-bargaining in social services) firmly established on one scale cannot be fully understood without considering their interrelations and interdependencies with political, economic and social orders on different scales, such as the local, regional or transnational levels. Hence, drivers of change in wage-setting institutions cannot be *a priori *expected on a particular spatial scale but instead their identification requires theoretical and methodological openness (Fibich et al. 2016). Both market liberalisation and countermeasures can unfold on different societal levels.

This article has the following structure: Sect. 2 builds a conceptual framework to guide the empirical research and evaluation. Sect. 3 provides a brief description of the social services sectors in Austria and Germany. The next section (Sect. 4) focusses on actors that partake in the historical and current struggles over wage-setting institutions and practices on various spatial scales. Of particular interest in this regard are the boundaries and spatial reach of evolving and established wage-setting institutions. The last section (Sect. 5) discusses the evidence in the light of the theoretical framework and draws conclusions.

## Conceptual framework

This article focuses on endogenous processes and social actors within and across certain fields that might have created the conditions for institutional change. Local and national actors might facilitate or inhibit the opening of markets; they may also initiate or oppose the worldviews of economic liberalisation and austerity predominating within the EU (Lamping 2008) and thus help (re-)structure European and global market structures and the social and symbolic orders. For example, while German public policy makers established market competition in certain sectors (e. g. hospital sector) long before European ministers agreed to make the EU the world’s most competitive and most dynamic economy, some consider the Lisbon Strategy 2000 as an impetus to liberalise the German labour market via the Agenda 2010.

However, research on employment relations and wage-setting institutions has derived key insights from cross-country comparisons and from exploring national responses to exogenous challenges (Traxler et al. 2001; Gumbrell-McCormick and Hyman 2013). Most recent industrial-relations research on European wage issues refers to the far-reaching transformation of national collective bargaining systems induced by the crisis politics and austerity measures of the EU and the Troika (the European Commission, the European Central Bank, and the International Monetary Fund) (Marginson and Weltz 2014; Meardi 2014; Lehndorff 2015). The EU’s new system of economic governance particularly marks a “paradigm shift in the EU’s approach to collective bargaining, from the acceptance of free collective bargaining to direct political intervention in national bargaining outcomes and procedures” (Schulten and Müller 2015, p. 331). New modes of disorganised decentralisation have partially replaced the once dominant logic of multi-employer collective bargaining.

To complement those institutional perspectives that highlight external drivers of institutional change, this article focuses on endogeneity: the local, regional and (trans-)national actors and institutions that help make, shape or dissolve collective bargaining practices and how their behaviour has repercussions on transnational markets and symbolic orders. Economic liberalisation as well as countermeasures taken by specific “societies” with particular traditions can drive institutional change and continuity (Streeck and Thelen 2005, p. 4). These processes primarily result from material and symbolic conflicts within and across interrelated social fields (Bourdieu and Wacquant 1992; Fligstein 2001). Conflicts in wage-setting fields might derive from various sources, such as workers trying to protect their rights, businesses shielding production and services against competitors or governments imposing austerity measures that affect power relations between employers and workers. Wage setting usually takes place at the intersection of markets and political-administrative fields, and it is an empirical question whether industrial relations fields with distinct wage bargaining practices and boundaries can be created and sustained.

If forming, reproducing and changing the wage-setting institutions are social processes that involve conflicts over resources and power relations in specific arenas of action or social fields (Bourdieu and Wacquant 1992), two decisive questions arise: Who benefits or loses from specific wage-setting institutions? And which actors are able (resource endowment) and willing (problem perception, dispositions and interests) to create, reproduce or change these institutions?

Collective actors who occupy powerful positions and benefit most from current field configurations are often referred to as “incumbents” and those who benefit less are “challengers” (Fligstein 2001). The structuration and restructuration of fields thus reflect power relations among the agents engaged in struggles over both the distribution of certain capitals (such as profits and wages) and the very definition of the most legitimate form of power in a particular field (such as economic power, political power). The latter is a struggle for symbolic power, a classification struggle (Swartz 2013, p. 58). For example, current labour law or collective bargaining practices reflect historical conflicts over working conditions and income distribution between capital and labour. Thus, the institutional outcomes of labour struggles themselves have become a legitimate form of capital: institutional power (Pernicka and Glassner 2014). These institutions contribute to shaping the behaviour and consciousness of field actors and, at the same time, they are constantly contested (Fligstein 2001). This conceptually powerful dialectic, however, leaves the empirical researcher with a significant question: which dynamic (institutional continuity or change) is more likely to occur and why?

Recent concepts within sociological neo-institutionalism (Zietsma and Lawrence 2010) and early conflict theory (Coser 1956) consider the (historically created) field boundaries and practices as a decisive element in analysing continuity or change. Practices refer to shared routines or customary rules;^3^ whereas, field boundaries denote the distinction between the outsiders and those insiders who share a common understanding of the practices and meanings of the “game” (Bourdieu and Wacquant 1992). Stable practices and field boundaries, for example, might induce business actors to continue with multi-employer bargaining even though weak trade unions could not force their participation. In contrast, weak or evolving fields with less stable boundaries and practices could create struggles over the very definition, scale and scope of the game. Two particular processes are important here: institutional and actor-related processes (see Table 1). In relatively stable fields, socialisation and adaptation processes more likely reinforce existing patterns of behaviour. Conflicts are largely confined to goals, values or interests that do not contradict the field’s founding assumptions. In comparison, open conflicts over the institutional setup become more important when long-standing field boundaries are compromised and existing power positions challenged. For example, when new actors, such as transnational companies, can enter local or national fields of wage bargaining, they might have the power to restructure them. Otherwise, if field boundaries and practices are stable, these former outsiders might take positions aligned with existing norms.

**Table 1:** Institutions and actors in the (re)structuring of wage-setting fields

Relatively stable fields of wage setting with relatively clear boundaries	Weak or evolving fields of wage setting with (yet) unclear boundaries
*Institutions*	
Regulative coercion, shared norms and cultural reproduction reinforce existing patterns	Contradicting rules, norms and cultural-cognitive understandings challenge existing practices of wage setting
*Actors*	
Institutionally embedded forms of conflict and cooperation between actors contribute to social reproduction rather than change	Power struggles over the “rules of the game” such as the forms and functions of wage-setting institutions and practices

The following sections concentrate on field boundaries and practices by presenting empirical evidence on the institutional and socio-economic structures and wage-setting institutions in social services as well as on actors, their positions and strategies. The empirical findings are mainly derived from 21 interviews^4^ conducted between April 2015 and March 2017. Twelve interviews were conducted in Austria, with representatives of unions, social service providers and their employers’ association (see Table 3 in the appendix). In Germany, nine interviews were conducted with representatives of unions, private and public employers’ associations, welfare organisations and service providers (see Table 4 in the appendix). The aim was to investigate the perceptions, dispositions, interests and behaviour of representatives of those organisations (potentially) involved in collective bargaining and wage setting in the social services sector. For Germany, Evans et al. (2012, p. 26) identify eight different arenas of collective bargaining, which are constituted by the six welfare associations, the public employers and the private for-profit employers (see below). All of these arenas encompass service providers from various subsectors. The interviews with representatives of service providers provided anecdotal evidence on Europeanisation processes in the sector. The interviews were transcribed and analysed using categories derived from theoretical reflections and examinations of the empirical material. The categories included institutional as well as associational and structural resources, practices and beliefs on various spatial scales, and inter- and intra-organisational relations, perceptions, interests and strategies of the actors. The interview texts were coded according to these categories. To validate the results, one researcher coded an interview and then another reviewed it. This qualitative-interpretative method allows reconstructing the historically created practices and social fields of wage setting in social services. The focus on a period from the mid-1990s until 2016 allowed the observation of some critical changes of wage-setting institutions and their driving social forces. Particular attention was given to the interrelations between market, political and industrial relations fields.

## Social services sectors in comparison

### Market and political fields in social services in Austria

Institutions and practices in the social services sector evolved according to local and regional rather than national power relations between political actors and service-providing organisations. Because of the subsidiarity principle that favoured private vis-à-vis public organisations, the regional governments began, in the late 1960s and early 1970s, a large-scale transfer of support to private welfare organisations providing social services. Social services in Austria are funded and regulated, to the largest part, at the provincial level (*Länder*) and Austrian federalism has promoted the emergence of varying regional “welfare cultures” (Dimmel and Schmid 2013, p. 53). Several political reforms shaped the funding of social services. Since the mid-1990s, public policy enforced competition and cost-cutting in the sector (e. g. by new forms of cooperation between private service providers and public agencies, competitive bidding procedures). Most European countries, including Germany and Austria, followed the trend of replacing direct subsidies to service-providing organisations with individual subsidies of clients using the services and with case-based compensation (Dimmel and Schmid 2013; Evers et al. 1994). To address rapidly increasing costs incurred by growing demand for care services, a national care fund with the aim to relieve municipal and regional social budgets was created in 2011. Specific funds were designated for long-term and old-age care.

In Austria, social services have been provided by public, private non-profit (churches and welfare organisations) and for-profit organisations. In 2014, 55% of elderly care homes were run by public entities, 25% by private non-profit and 20% by private for-profit organisations (Knight Frank Research 2014). With the partial exception of old-age care, where the number of privately run care homes has increased while the number of public care homes declined, service provider structures have remained stable in Austria since the early 2000s (Interview 04; Nam 2003). In comparison with Germany, Austria has more limited access for transnational companies to the highly regulated (and thus, supposedly less attractive) market. Two international social care groups run elderly-care homes in Austria, with notable differences between regions. Other subsectors have a small presence of transnational companies, although some subsectors had internationally competitive tenders in areas such as workforce training and further education as well as patient transport.

Employment in social services has steeply increased from 2004 to 2017. While around 81,500 employees were recorded in social services in 2004, this number rose to 128,600 in 2014. Relative growth in social services exceeds average employment growth. The share of female employment (75%) and part-time work (45%) has been high (Statistik Austria 2015). Most sub-sectors and occupations have not lacked qualified staff. Wage levels in social services have been around 20% below average gross earnings of all employees in Austria (Dimmel and Schmid 2013).

### Market and political fields in social services in Germany

Germany’s federal constitution mandates that municipalities fund and provide social services. However, since the Weimar Republic, a principle of subsidiarity largely delegates the particular tasks to non-profit and—increasingly—commercial providers (Bäcker et al. 2010). The funding for municipal tasks rests on three pillars: taxes, which are mainly subject to federal legislation, redistribution of income between levels of the federal state, and fees and dues (Vesper 2015, p. 55). Not all social services, however, are funded by the municipalities; some are funded by social insurances, such as long-term care for which a statutory insurance was established in the mid-1990s.

In the non-profit sector, six welfare associations provide most services. The confessional associations are Caritas Germany (Catholic), Diakonie Germany (Protestant) and the Zentralwohlfahrtsstelle der Juden in Deutschland (ZWST)^5^. The secular organisations include Arbeiterwohlfahrt (AWO)^6^, the Paritätische^7^ and the Deutsches Rotes Kreuz (DRK)^8^. However, the structure of providers varies widely between sub-sectors and regions. An estimated 42% of all social services in Germany are delivered by non-profit providers, 33% by public providers and 23% by private for-profit providers (Merchel 2011, p. 245). Especially in elderly care, private for-profit providers have become significantly important. In the area of care homes, for-profit providers ran 41% of all facilities in 2014 (54% non-profit, 5% public); in the area of mobile care services, for-profit providers account for most facilities (64%) (Statistisches Bundesamt 2015). Besides providing services of general interest, the social services sector in Germany has great economic importance. It accounts for 7% of regular employment; the welfare associations’ share of value added compares with that of the chemical industry (Kühnlein and Wohlfahrt 2006, p. 390). Over 2.2 million people have been employed in the social services sector in 2016. This means a 4.3% growth compared to the previous year (Bundesagentur für Arbeit 2016a). The average monthly earnings in the social services sector are an estimated 10–15% below those in the rest of the economy (Evans et al. 2012). Since 2010, a statutory minimum wage exists for care personnel in care companies. All interview partners perceived a shortage of qualified personnel (see also Bundesagentur für Arbeit 2016b).

## The contested evolution of wage-setting institutions and practices in social services

### The creation of the national sectoral employers’ association in Austria

For Austria’s social services, new institutions and practices of wage setting at the national level reinforced a social field. This section will describe the formation of a national employers’ association with collective bargaining rights that then decisively shaped the social field. The next section will trace the historical struggles over the spatial scope and scale of field boundaries and practices and, in particular, how regional and local political actors opposed a nationwide collective agreement. This section’s final part considers the current state of field boundaries and practices and their impact upon the perceptions, interests and behaviour of field participants, including transnational social service providers.

Austrian social service employers formed two important organisations. In 1995, the “Bundesarbeitsgemeinschaft Freie Wohlfahrt” (BAG) was founded to represent the five largest private non-profit providers as a lobbying organisation but not to conduct collective bargaining. Against the background of increasingly widespread public procurement and outsourcing by regional governments, employers aimed to establish their own “voice” vis-à-vis political actors (Interviews 05 and 13). Further negotiations on creating a distinct employers’ association with the explicit aim to negotiate a national sector-wide collective agreement had started in the early 1990s. Finally, after protracted negotiations (see Sect. 4.2), five provider organisations created the “Berufsvereinigung von Arbeitgebern für Gesundheits- und Sozialberufe” (BAGS) in 1997. However, some large providers, such as the Red Cross and church-related organisations, did not join.

Longstanding, close personal relations between employers helped create the BAGS association. In particular, some key actors held dual (professional) roles in the employers’ organisations and the management of social service organisations. Historical linkages between political parties at the provincial and local level and certain, often large, providers limited competition and resulted in informal allocation of (parts of) service markets. Throughout the 1990s, these relations became less relevant due to the abolishment of the government’s funding system based on proportional support to providers related to political parties (Interviews 05 and 13).

The BAGS gained the legal right, in 1997, to conclude collective agreements for the private (non-profit and for-profit) providers of social services because the responsible state authority (das Bundeseinigungsamt^9^) considered the organisation to be of “considerable economic relevance” in the sector due to its representativeness of employees in the sector. Trade unions supported the legal process that resulted in the BAGS associations’ entitlement to collective bargaining: “Because we also wanted the capacity to conclude collective agreements, we supported employers and we were in the senate of the Federal Arbitration Board” (Interview 13). The Austrian Caritas organisation, as the largest employer in social services (around 14,000 employees in 2015), also gained the right to conclude organisational level collective agreements, alongside other organisations such as the Austrian Red Cross and Diakonie.

Employers agreed that it would be in their interest to harmonise pay and working conditions and replace the highly fragmented and non-transparent system of collective agreements from outside the sector (e. g. commerce), and a patchy set of legal regulations on minimum wages and working conditions not covered by collective agreements. Their perceptions and interests seem to be particularly influenced by the wider institutional environment in national fields of collective bargaining that has firmly established norms and ideas of cooperation and covers almost 100% of the workers (Pernicka and Hefler 2015). In addition, employers articulated a particular interest in flexible working times that deviate from legal norms. This can only be achieved by concluding a collective agreement (Interviews 04, 06 and 13). The planned “eastern enlargement” of the EU had raised fears of the inflow of cheap labour that would reduce wages and that could only be countered by a national sectoral minimum wage (Interviews 03 and 12). Furthermore, employers wanted to avoid being played-off against each other in negotiations with regional social service funding sources. Finally, employers agreed on the need to recruit well-qualified workers and increase the attractiveness of social service professions for young people. Also, national harmonising and recognition of professional qualifications would enhance the workers’ geographical and professional mobility. Trade unions also largely shared these goals and interests. Trade union and employers representatives acknowledge that the BAGS agreement resulted from the shared understandings and the aim “to commonly create something big, something good for all” in the sector (Interview 02). They also emphasised that such a constellation of forces would no longer be feasible because of the increased importance of cost competition and austerity policies (Interview 13); the current market logic hinders practices of nationwide cooperation and coordination in wage setting.

### Struggles over and counterforces against a sector-wide agreement in Austria

The negotiation of the BAGS-collective agreement took more than six years and resulted from protracted struggles between social partners that formed an inter-class coalition, on the one side, and political actors, on the other side. In addition, conflicts arose within the labour and employers’ camps. However, the fiercest opposition against a national sectoral agreement came from regional governments arguing that they could not fund a nationally harmonised system to pay a wide range of social professions. Government representatives openly preferred to improve their bargaining position by negotiating separately with individual providers. Employers’ determination to end such divisive tactics inspired their support of the national sectoral agreement (Interviews 03, 04 and 05). Interview partners note the continuance of austerity policies that are, however, not directly linked to the European fiscal and debt crisis.

Conflicts and struggles also took place among employers’ organisations. The Austrian Economic Chamber (Wirtschaftskammer Österreich, WKÖ) represents all Austrian companies, for which membership is obligatory. They opposed setting up a sectoral employers’ organisation with voluntary membership that has a higher priority in collective bargaining than organisations with compulsory membership. Recently, even not-for-profit companies became members of the WKÖ; however, most also belong to BAGS which continues to grow since its founding. Currently, BAGS (renamed into Sozialwirtschaft Österreich, SWÖ^10^, in 2012) organises 367 member organisations which employ more than 55,000 workers in private social service provision (SWÖ 2016). Organisational density of employers, i. e. number of workers employed in BAGS/SWÖ member organisations in relation to all employees pertaining to the organisational domain of the association, is approximately 55%.

On the side of labour, two trade unions conclude collective agreements in social services: the white-collar workers’ union GPA-djp, which is the largest union in Austria, and the vida union, which operates in industrial and transport sectors. Because their organisational domains partially overlap, the two trade unions coordinate their membership policies in order to avoid competing for members (Interviews 02, 06 and 13). Trade union density in social services is approximately 18% with wide variation between subsectors (Interviews 02 and 13). Despite this relatively low associational power of trade unions, collective bargaining coverage in Austrian social services is approximately 95%.

Within the social services sector, the BAGS agreement, covering the largest part of employees in the sector, serves as a pattern-setting agreement for other social services organisations. However, this pattern setting has an indirect effect; large providers state that they do not explicitly refer to pay rates settled in the BAGS agreement (Interview 15). Instead, the national agreement serves as an informal, implicit, but still effective, guideline in single-employer bargaining with large social services organisations. Most employers closely follow the negotiation of the BAGS agreement and its outcomes and follow the wage increases stipulated in the national sectoral and the company agreements of large social service providers. Representatives of the SWÖ and some of the largest service providers once attempted to jointly negotiate the national sector-wide agreement and the company agreements of organisations such as Caritas and Diakonie (Interviews 06 and 15).^11^ Although the attempt failed, employers continue to informally coordinate with each other in collective bargaining, indicating the existence of a field of wage setting in social services that extends beyond formally established practices of collective wage bargaining. Over time, the SWÖ agreement became relevant beyond the private social services sector. For example, a recently negotiated public sector pay system in hospitals and residential care in the Upper Austria province is oriented towards the SWÖ agreement (Interview 01). The new remuneration system for public employees in the Lower Austria province also refers to the SWÖ agreement (Interview 06).

At the same time, collective bargaining in social services became more independent and autonomous from wage setting in the public sector. Bargaining actors state that they “do not care about the public sector”. That’s a “big taboo” in negotiations (Interview 13). In fact, however, they closely observe the public sector’s pay negotiations, which always take place before collective bargaining in social services, and use the results as a benchmark: “We have always achieved pay settlements approximately as high as in the public sector” (Interview 04) or even “above the public sector” (Interview 02).

Even when interview partners were explicitly asked if actors or agreements in other countries or at the European level affect their own positions, they responded that these international events do not influence their negotiating. Despite the opening of national social services and labour markets, wage-bargaining fields do not extend beyond national boundaries. In certain segments of social services, such as stationary old-age care, transnational companies have entered the Austrian market; however, they have limited influence on service and labour markets and must accept the national sectoral agreement. Wage-bargaining actors in Austria successfully created particular normative orientations towards sector-specific views on collective bargaining; hence, even representatives of transnational social services providers adopt these views. For example, after SeneCura (part of the global Orpea corporation) opened operations in Austria, it applied the BAGS agreement and joined the sectoral employers’ association. The Austrian-led management cooperates with trade unions. This situation contrasts to other countries, such as France and Belgium, with escalating conflicts between trade unions and the Orpea management (Interviews 02, 06 and 13). The emergence of a largely autonomous field of wage bargaining in social services was fostered by socialisation of field participants towards coordination and cooperation, their power relations and the formation of behavioural and legal norms.

Despite the relative autonomy of the collective bargaining field in Austrian social services, actors feel the impact of European level developments. Both trade unions and employers closely observe policy making in the European Union and try to actively influence it via lobbying on issues such as revising the public procurement directive. They feel that this directive and other EU regulations, such as on state subsidies, indirectly influence the provision of social services in Austria. The EU directive on public procurement leaves considerable leeway in implementation by policy actors. Its use and interpretation varies widely between Austria’s provinces. Trade unions and employers note the increased importance of public bidding and procurement in certain subsectors such as labour market policies, care of disabled people and patient transport services (Interviews 04 and 10). Both trade union and employer representatives appreciate that the directive’s recent revision places more importance on quality criteria vis-à-vis cost considerations. However, they criticise the large administrative burdens, particularly for smaller providers, to prove quality standards (Interviews 14 and 15).

Austrian social partners also promote coordination and organisational structures transnationally. For example, vida and GPA-djp as well as SWÖ representatives participate in PESSIS, a European Commission project initiated in 2012. The project, expected to run until late 2017, seeks to sound out possibilities for European social dialogue in social services. The most important obstacle to European social dialogue is the lack of sectoral employers’ organisations in most European countries.

### Developments of wage setting in German social services

In contrast to Austria, collective bargaining in German social services has fragmented. The formerly established rather “weak” field of wage bargaining coordination based on actors orienting themselves towards the public sector collective agreements seems to be eroding. Some authors state that public sector agreements have already lost their function as the reference point for wage regulation in social services (Kühnlein and Wohlfahrt 2006, p. 392; Grohs and Bogumil 2011, p. 309). Actors in the field take different stances towards collective wage bargaining (coordination), ranging from trade union efforts to (re-)establish sector-wide collective bargaining to private companies operating without any collective regulation of their employment relations. New policies undermined rather than facilitated the creation and reproduction of coordinating institutions and practices of wage bargaining in German social services. Therefore, the following sections outline some important political and administrative developments on the national and regional levels. The next section presents the fragmented and patchy pattern of wage-setting practices and the still ongoing struggles over the legitimacy and value of collective bargaining in social services. Because employers, i. e. social services providers, have perceptions, interests and behaviour that support competitive rather than cooperative practices to determine wages, these field participants will be described in more detail.

Legal reforms and new forms of funding social services were implemented to increase competition between providers. Many actors in the sector mention the decisive importance of the legislation in 1995 on long-term care insurance, because the reform boosted competition and strengthened the role of private for-profit providers of care (e. g. Interviews 20 and 21). The reform in long-term care had aspects that also served as a model for reforms in other parts of the sector (Buestrich and Wohlfahrt 2008, p. 20; Interview 17). Also, regulations and practices concerning the funding of social services vary by subsector and region and have a decisive impact on the sector, e. g. on the structure of providers (e. g., caps on expenses for care places may disadvantage providers with higher wages). Union representatives see a general problem in that funding social services depends on the public authorities’ resources and—in the case of some services—the statutory social security. With austerity politics, providers and employees in social services face a constant pressure to cut or at least curb costs and unions criticise that cost-efficient service provision is given higher value than good working conditions for the employees (Interviews 19 and 22).

The state authorities do not unilaterally fix the rates and framework conditions (e. g. staffing levels) for the delivery of social services; they negotiate with associations of service providers mostly at the level of the states (Bundesländer).^12^ Also, the actors can lobby and exert some influence in policy making. For example, both unions and welfare associations view it as a success that the law on the long-term care insurance now states that collective agreements may not be considered “inefficient” when reimbursing social service providers. However, this can be partially counteracted. Some clients require additional social benefits to pay for their care place. These must be paid by the municipalities, who may try to place clients in facilities with lower costs. Because the municipalities must pay social benefits, they also play an important role when fixing the reimbursement rates for social service providers. They seek to keep costs low, because they must cover clients not able to pay for their care place (e. g. Interview 16). Unlike Austria, German service providers did not seek to establish ongoing cooperation to counter pressures from funding authorities. The fragmentation and non-cooperation among employers seem to be the main hindrance in the sector to more encompassing regulation of employment conditions and coordination of wage policy. Representatives of all employers’ associations see certain overlaps of interest and they occasionally cooperate and exchange information (e. g. in the minimum wage commission or in negotiations on renewed funding with state actors). However, they do not perceive stronger coordination as possible or even desirable. The open competition between providers, even within the same association, hinders cooperation (Interviews 19 and 17). Most provider associations embrace the logic of competition promoted by political actors. Because the German social services market has fewer regulations and promises high profitability, it also seems more open and attractive to transnational corporations than the Austrian one (Interview 21). However, most interview partners agree that competition from multinational companies does not add a different quality to the already existing competition in the German social services market.

EU regulation does not appear to directly influence the German social services sector because the national regulatory framework mediates EU directives. However, unions as well as employers’ and welfare organisations lobby in the EU policy-making process, e. g. in revising the public procurement directive. Also, EU regulation on public subsidies has an impact on the German social services field. Private providers have tried to use these regulations to restructure the German market in their favour and addressed complaints to the European Commission; however, from their point of view, the results were unsatisfactory (Interview 24). Non-profit providers see constant relevance in EU regulations and a source of uncertainty as to whether their funding advantages may conflict with EU limits on public subsidies (Interviews 20 and 21).

### The fragmented landscape of wage-setting institutions in German social services

The German social services sector’s most encompassing organisation is the Vereinigung der kommunalen Arbeitgeberverbände (VKA)^13^. It covers the public municipal part of the sector with an organisational density in its domain of close to 100%. The VKA concludes the collective agreement for the public sector (TVöD) with the Vereinte Dienstleistungsgewerkschaft (ver.di)^14^, which is by far the biggest union in the social services sector. Another smaller union, the Gewerkschaft Erziehung und Wissenschaft (GEW)^15^, has relevance in the sector, such as the area of kindergartens.^16^ The social services sector, as compared to other sectors, has a low union density of approximately between 3 and 10% (Evans et al. 2012, p. 30). The particularly low union density among church employees has an impact on the overall union density in the social services sector. However, ver.di’s social services division has increased membership.

The church-related providers Caritas and Diakonie enjoy the special right to conclude guidelines for employment contracts via the so-called “third way”. This means that special commissions staffed with employee and employers’ representatives negotiate these guidelines. However, in some of its regional divisions, the more decentralised Diakonie has diverted from this third way and concluded collective agreements with ver.di. Many diaconal enterprises are represented in the Verband diakonischer Dienstgeber in Deutschland (VdDD)^17^, founded in 1996 to promote the interests of diaconal enterprises who felt that the religiously oriented Diakonie did not represent well their business interest (Interview 17). VdDD representatives principally favour competition between social service providers—or feel that it is inevitable, because reimbursement of providers for all costs incurred is believed to be a thing of the past; however, they demand the same framework conditions for all competitors. These framework conditions should, in their view, include reimbursing costs that result from higher wages if a collective agreement binds a provider. The VdDD aims to create an encompassing framework of collective regulation for all diaconal enterprises in Germany. In order to compete with private for-profit providers who operate without collective agreements, this framework would offer the possibility for differentiated wages in different regions and subsectors (Interview 18). The VdDD also has the explicit aim to move away from the TVöD, since the structure and “ideology”^18^ of the TVöD would not fit the business needs of its members (Interview 17).

Caritas-related enterprises also have a separate organisation to represent their entrepreneurial interests. However, the Caritas adheres to the “third way” more strictly than the Diakonie and it also orientates its employment regulations more closely to the TVöD than the other welfare associations (Interviews 25 and 26).

While the AWO has its own employers’ association, the Paritätische and the DRK have so-called (regional) collective bargaining communities. This, however, does not mean that the welfare associations have coherent collective agreements or guidelines for salary and working conditions that cover all their facilities. Instead, there is a fragmented landscape of regional, local and company collective agreements (if an agreement is concluded at all) (Interview 19). All of these welfare associations have decentralised structures and weak peak level governance, which allows the single facilities or regional associations to act relatively autonomously when organising their employment relations (Interview 19). A union representative noted the self-made origins of the associations’ decentralised structures. The AWO dismantled their national collective agreement in the 1990s (Interview 19). In the 1980s, several unions helped with a failed attempt to establish an employers’ association for the Paritätische in order to try negotiating a collective agreement (Interview 22). Even though some actors within the welfare associations again favour more coordination in collective bargaining, this increased cooperation could not easily be (re-)established (Interview 19).

The two for-profit employers’ associations in the sector, the Arbeitgeberverband Pflege (AGVP^19^, founded in 2009) and the bpa employers’ association (breaking away from AGVP in 2015) have obvious differences but share the characteristic of weak peak level structures to some extent. These associations represent a diverse membership of over 1700 facilities in the social services sector (Interviews 16 and 24). Both private employers’ associations were mainly founded to influence the minimum wage legislation in care via the care commission^20^ and not necessarily to conclude collective agreements. However, in the summer of 2016, the AGVP did plan to negotiate with ver.di concerning a collective agreement for apprentices. However, due to internal changes within the AGVP, negotiations have not yet started. The bpa employers’ association is working on its collective bargaining strategy. Both associations strictly oppose generally binding collective agreements (Interviews 16 and 24). The AGVP’s steps toward negotiating a collective agreement with ver.di could thus possibly be seen as a strategy to counter ver.di’s (and the GEW’s) attempts to conclude (regional) collective agreements with bargaining communities of different provider associations and have these declared generally binding. However, this strategy has not been very successful (Interview 19). The fierce competition between facilities in a fragmented provider landscape in combination with the restrictive funding makes it hard for ver.di to conclude collective agreements with single providers who fear to lose out in competition (Interview 19). Because the sector’s wage levels are very diverse and sometimes well below that of the TVöD, the union sometimes concludes collective agreements below TVöD-standards and then tries to raise wage levels over time. VKA sees this as a problem; such agreements could weaken TVöD and the municipal providers adhering to it (Interview 23). Ver.di acknowledges this problem and makes great efforts to transfer public sector standards to private for- and non-profit providers despite the increasing difficulties according to ver.di (Interview 19). Overall, collective bargaining is very diverse and fragmented in the social services sector. Evans et al. (2012) have counted over 1400 collective agreements and guidelines for employment contracts in the sector. They note that collective regulation does not cover 63% of the facilities and 37% of the employees. Especially with the sector’s private companies, up to 80% of employees are not covered by a collective agreement (Evans et al. 2012, p. 29 f.). Private providers conclude collective agreements mainly at the level of single companies or facilities, if at all (Interview 16). Despite this, a public employers’ representative holds that the TVöD still has a big influence on wages in the sector.^21^ The confessional associations, especially Caritas, would strongly orientate themselves to the TVöD. Other non-profit providers would also adhere to the TVöD, at least concerning basic provisions of the collective agreement (Interview 23). Most interview partners agree that private for-profit providers do not follow the public sector’s lead concerning wage levels, but use the TVöD as a reference point for reimbursement of services by the funding authorities. The resulting gap between reimbursement and wages paid provides them with entrepreneurial freedom (Interview 17). However, the private employers’ associations see the TVöD as a reference point to some extent and hold that private care companies do not pay (much) lower wages than other providers because of the fierce competition for qualified staff (Interviews 16 and 24). The TVöD also has strongly differing significance according to subsectors because the share of service providers located in the public sector bargaining arena differs between subsectors (Interview 18).

As in Austria, most of the German employers’ associations have participated in the EU’s PESSIS project. However, most employers’ associations take a cautious stance on the project. Despite exploration of closer cooperation between employers’ associations, most interview partners do not see much progress. Some also explicitly state that closer cooperation should not include the area of collective bargaining; they see the PESSIS-project as a chance for social services enterprises to be heard at EU-level with their business interests (Interview 17).

## Discussion and conclusions

This article set out to better explain the (re-)structuring of employment relations and wage-setting institutions in social services in Austria and Germany. It focused on the contested nature of labour market institutions and the specific conflict dynamics within and across social fields. Diverse societies organise and reorganise their labour, product and services markets according to the results of conflicts over sets of rules, norms and ideas. These conflicts have more effects on social distribution than the mere outcome of efficiency-seeking actors in competitive markets or of institutionally determined trajectories. The research has been guided by the following assumptions: Even if economic, political or social events evolve outside a specific field, such events are mediated and implemented (if at all) contingent on the field boundaries and practices as well as on the dynamics of conflicts and cooperation between the varied actors. Thus, contingent on the state of (historically created) fields, various groups of business and labour actors as well as governments and administrative bureaucracy continually reproduce or change institutions. In following Bourdieu’s conception of social field, this article has sought to reconstruct wage-setting fields in social services based on research of power relations, positions and strategies pursued by involved agents. In both Austria and Germany, the similarly organised provision of social services has a triangular relationship between the state (funder), autonomous providers and individuals who receive the social service. Both societies have faced the opening of national labour markets for eastern European workers and provide possible (!) markets for private multinational companies that provide social services. Furthermore, actors in both fields of social services have seen the European Commission treat social services as services of general economic interest, which makes them subject to EU competition law.

However, despite the similar pressures by European and international political and economic forces on Austria and Germany, their employment relations and wage-setting institutions turned out to follow different institutional paths. In Austria, the interdependencies and interrelations between actors at multiple spatial scales shaped these actors’ power resources and positions and contributed to the emergence of a relatively autonomous collective bargaining field. A nationwide field of wage setting in social services in Austria could be created despite fierce opposition from the field of regional public authorities, a field which had actors that feared losing competence and power, as well as employers struggling over relevant competences. In comparison with Germany the field of collective bargaining still remains relatively closed to European and transnational influences.

By contrast, social services providers and workers in Germany have experienced the disruption of a weakly established field of wage bargaining with actors oriented towards public sector agreements. They face highly fragmented and institutionally segmented structures of wage determination including local and regional differences in wage negotiating, statutory minimum wages and companies operating without any collective regulations of their wage-setting practices. How can these differences be explained?

Based on Bourdieu’s theory of social fields, this article has emphasised the role of social actors and their (institutionalised) power relations in creating practices, norms and beliefs of wage-setting fields in social services. In particular, the article asserted a dialectical interrelation and interdependency between institutions and actors in the historical trajectories of wage-setting practices. If wider institutional configurations and field boundaries are relatively stable, prevailing patterns of behaviour are more likely to be reproduced and even extended to newly created sub-fields rather than changed. The opposite is likely to occur in evolving and less mature fields in which open conflicts over the boundaries and practices could prevail (see also Table 2).

**Table 2:** Comparing wage-setting fields in social services and their creation in Austria and Germany

Austria	Germany
*Provider structures*	
Social services provision by private for-, non-profit and public organisations; emergence of large transnational corporations in certain subsectors such as elderly care; German market more open to transnational firms than Austria	
*Political relations*	
European Commission wants to enhance market logics in social services National governments have reformed funding regulations for social services (e. g. direct subsidies to service-providing organisations replaced by individual subsidies of clients using the services and case-based compensation). These measures occurred in the context of the politics of public austerity, cost-cutting and competition since the mid-1990s. Regional (Länder) and municipal authorities who administer social services provision have come under increasing cost pressure	
*Wage-setting fields*	
A nation-wide, collective wage bargaining field could be established by an inter-class coalition consisting of BAGS and trade unions in the 1990s despite fierce opposition of regional governments	A weakly established field of wage bargaining coordination was disrupted and gave way to highly fragmented, institutionally segmented structures and practices of wage determination
*Facilitating and impeding institutions*	
The relatively stable state of national wage bargaining fields helped create an autonomous subfield in social services. Highly legitimate norms and values of (cooperative) conflict resolution and multi-employer wage bargaining could be transferred to social services	The prevailing logic of market competition primarily promoted by actors in the German political field since the 1990s also spread to wage-setting fields and has undermined the legitimacy of cooperative forms of wage setting
*Actors and power-relations*	
Close and longstanding personal relations between employers in social services who formed the employers’ association BAGS and trade unions backed by national administrative bodies paved the way for an inter-class coalition that reduced the role of the previously dominating regional governments in wage setting	Actors in favour of coordination (unions, some welfare associations) were weakened by the large heterogeneity of structures and processes of wage setting on regional and municipal levels as well as by prevailing logics of competition enhanced by political actors, private for-profit service providers and some welfare associations

Austria was more likely to create a wage bargaining field in social services because institutional entrepreneurs on both sides of the employer-employee divide could utilise power resources provided by the wider institutional environment. Austria’s multi-employer collective bargaining in social services was facilitated by the exceptionally high level of collective bargaining coverage of almost 100% of all dependently employed workers in Austria (Pernicka and Hefler 2015) and the firmly established norms and ideas of cooperation in national fields of employment relations. The “partnership-driven approach” of employers on labour market and employment issues was a crucial force in creating favourable conditions for workers and avoiding competitive undercutting of pay and conditions. Furthermore, the aim to improve, vis-à-vis government actors, the negotiating position on the renewed funding of social services has promoted intra-class alliances among employers wanting to create a sectoral collective agreement. In contrast to most social services employers in Germany, Austrian employers seem still to be socialised to prefer negotiated, common solutions over individual strategies of wage competition. However, several interviewees note that if the context would have included the recently legitimised austerity policies, then regional public policy and administration actors would have been able to successfully mobilise against a national wage agreement.

German actors, in contrast, seem to have adapted to and internalised the logic of competition primarily promoted by those in the German political field since the 1990s. Evans (2016) points out that employers’ associations in social services were not founded in reaction to strong unions but created in order to shape working conditions distinct from those in the public sector and to promote business interests against the “charity” ideology of welfare associations. This was deemed necessary in reaction to prevailing governance modes of “market” and “competition” in social services (Evans 2016, p. 26). One might argue that, both in Austria and Germany, emancipation from public sector logics in employment regulation helped inspire the founding of employers’ associations; however, the path of emancipation led in almost opposite directions. The highly fragmented and patchy set of regulations embracing statutory minimum wages and collective agreements at different levels have contributed to very diverse wage levels in the German sector. The boundaries and practices of wage setting are subject to ongoing conflicts between employers and trade unions and also within trade unions and employer associations. The structuring and restructuring of social services markets and wage-setting fields have an impact upon industrial relations actors and income distribution at local and national level. The symbolic dominance of market liberalism, public austerity and authoritative wage policies within the EU meets local, regional or national fields that reproduce different institutionalised forms and practices of wage settings. Given the varying entanglements of political, economic and industrial relations fields within a highly integrated Europe, the way a society organises its employment institutions has repercussions on the structuration of transnational social space. In terms of the economic field, Germany has a far more open market of social services than Austria; this has consequences on the strategies and positions of multinational companies. In addition, the highly institutionalised fields of wage bargaining coordination in Austria might serve as a leverage to increase symbolic power within the EU, when it comes to establishing a European social dialogue in social services. Even if symbolic power relations in transnational public policy, market and industrial relations fields strongly support market logics over coordination, the conflicts over the boundaries and practices in wage setting are very likely to continue.

## Appendix

### Interviews

**Table 3:** Austria

No	Date	Level	Function anonymised
01	15.04.2015	Regional	GÖD, regional level
02	17.12.2015	National	GPA-djp, national level
03	22.01.2016	National	(Formerly) Volkshilfe Österreich, national level
04	01.02.2016	National	Sozialwirtschaft Österreich, national level
05	19.04.2016	National	Hilfswerk Austria, national level
06	23.05.2016	National	Vida national level
07	23.05.2016	National	Vida, national level
08	08.07.2016	National	Vida, national level
09	08.07.2016	National	Vida, national level
10	08.07.2016	National	Vida, national level
11	10.08.2016	National	Volkshilfe Österreich, national level
12	10.08.2016	National	Sozialwirtschaft Österreich, national level
13	11.11.2016	National	GPA-djp, national level
14	25.11.2016	National	Volkshilfe Österreich, national level
15	05.12.2016	Regional	Caritas Wien, regional

**Table 4:** Germany

No	Date	Level	Function anonymised
16	22.06.2016	National	Representative private employers’ association
17	20.07.2016	National	Representative diaconal employers’ association
18	20.07.2016	National	Representative diaconal employers’ association
19	10.08.2016	National	Ver.di representative
20	01.09.2016	Enterprise	Representative confessional social service provider
21	06.09.2016	National	Representative business association of non-profit providers
22	21.11.2016	National	GEW representative
23	25.11.2016	National	Representative public employers’ association
24	2.12.2016	National	Representative private employers’ association
25	13.3.2017	National	Representative employers’ side of the ARK of the Caritas
26	13.3.2017	National	Representative employers’ side of the ARK of the Caritas

## Footnotes

1 Even if the social services sector is closely interrelated with health services and the sector’s exact boundaries have remained contested, this article seeks greater clarity by focusing on the social services sector (for such an approach, see Evans et al. 2012). Differentiation between health and social services also seems justified when considering the practice of industrial relations at the European level: a sectoral social dialogue for hospitals is already established, a sectoral social dialogue for social services is still in preparation (for collective bargaining in German hospitals, see Glassner et al. 2015).

2 Collective wage bargaining refers to negotiating between and within groups of employers and employees with or without the participation of state actors; this bargaining seeks a collective agreement to govern employment. Contrary to uncoordinated setting of wages subject to market logic, collective wage bargaining is based on cooperation between organised business and labour.

3 Bourdieu’s concept of practice should be seen in the light of his attempt to overcome both structural determinism and subjective voluntarism. “It would thus be possible to move on to the ground where talk of rules seems least misplaced […] and show that the ‘customary rules’ preserved by the group memory are themselves the product of a small batch of schemes enabling agents to generate an infinity of practices adapted to endlessly changing situations, without those schemes ever being constituted as explicit principles” (Bourdieu 1977, p. 16).

4 Five interviews were conducted with two interviewees simultaneously present. Interview numbers throughout the text refer to individual interviewees.

5 Central Welfare Board of the Jews in Germany.

6 Workers’ Welfare Association.

7 Joint Association of Welfare Associations.

8 German Red Cross.

9 The Federal Arbitration Board.

10 Accordingly, the BAGS-agreement was renamed into SWÖ-agreement, hence both terms are used synonymously.

11 The Red Cross, as third largest service provider, refused to participate due to its highly diverse pay and remuneration systems that vary between regions and are often oriented towards public sector pay schemes.

12 Also, various corporatist arrangements still include non-profit providers.

13 Joint association of municipal employers’ associations.

14 United Services Union.

15 Education and Science Union.

16 There are also some small business friendly trade unions active in the sector, especially in eastern Germany, where they concluded collective agreements with some welfare associations (Evans 2016, p. 26).

17 Association of Diaconal Employers in Germany.

18 Reforms of public sector collective agreements notwithstanding, some still regard its provision as hostile to performance and efficient service delivery.

19 Employers’ Association Nursing and Care.

20 The care commission was first convened in 2009 to recommend a minimum wage in care which can then be enacted by decree of the Federal Ministry of Labour. The commission, when convened in 2017 for the third time, had two representatives from ver.di, two employee representatives from the church commissions, two employer representatives from the church commissions, one representative of the VKA and one representative from the bpa employers’ association.

21 The TVöD resulted from a conflictual reform of the public sector collective agreements in the first half of the 2000s. After implementing the TVöD, employment relations in some parts of the public sector, especially child care, became more conflictual (Schmidt et al. 2011).
